# Supplementary material for: A druggable conformational switch in the c-MYC transactivation domain
Source: Nat Commun. 2024 Feb 29;15:1865. doi: 10.1038/s41467-024-45826-7 (PMC10904854; doi:10.1038/s41467-024-45826-7)
Supplement: Supplementary file 1 — Supplementary Information [file 41467_2024_45826_MOESM1_ESM.pdf]

Supplementary Information

A druggable conformational switch in the c-MYC transactivation domain

Dilraj Lama[1,#,*], Thibault Vosselman[1,#], Cagla Sahin[1,2], Judit Liaño-Pons[1], Carmine P. Cerrato[1], Lennart Nilsson[3], Kaare Teilum[2], David P. Lane[1], Michael Landreh[1,4,*] and Marie Arsenian Henriksson[1, 5*]

[1]Department of Microbiology, Tumor and Cell Biology (MTC), Karolinska Institutet, Biomedicum, SE- 171 65 Stockholm, Sweden

[2]Structural Biology and NMR Laboratory and the Linderstrøm-Lang Centre for Protein Science, Department of Biology, University of Copenhagen, DK-2200 Copenhagen, Denmark

[3]Department of Biosciences and Nutrition, Karolinska Institutet, SE-14813 Huddinge, Sweden

[4]Department of Cell- and Molecular Biology, Uppsala University, SE-751 24 Uppsala, Sweden

[5]Division of Translational Cancer Research, Department of Laboratory Medicine, SE-221 00 Lund University, Lund, Sweden

[#] Equal contribution
*Address correspondence to:

Dilraj Lama, E-mail: dilraj.lama@ki.se
Michael Landreh, E-mail: michael.landreh@ki.se
Marie Arsenian Henriksson, E-mail: marie.arsenian.henriksson@ki.se

Contents

- Supplementary Figures 1-21
- Supplementary Tables 1-3
- Supplementary Note
- Supplementary References

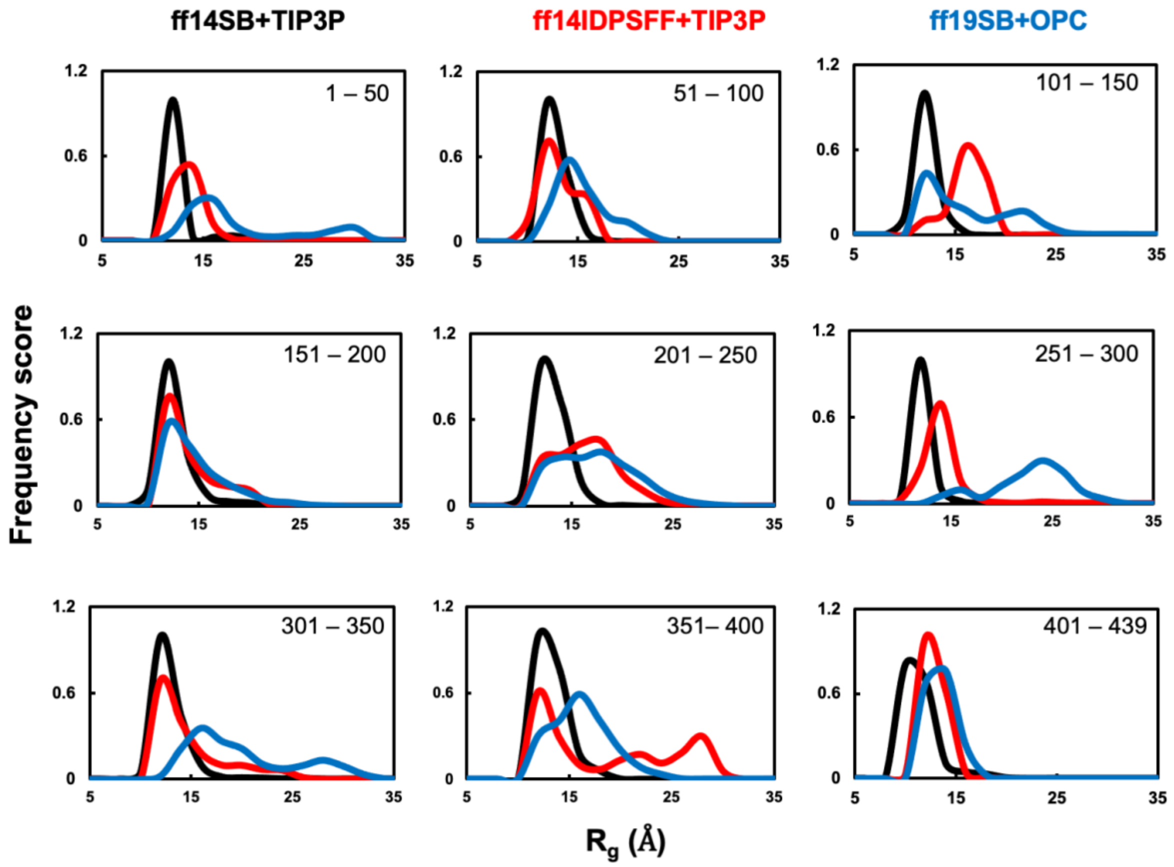

Supplementary Figure 1. Assessment of force-fields and water models. Normalized frequency distribution of R_g computed from the ensemble of structures generated from 1μs explicit solvent MD simulations for the library of nine non-overlapping c-MYC peptides in three different combinations (ff14SB+TIP3P, ff14IDPSFF+TIP3P, and ff19SB+OPC) of AMBER-based protein force-fields and water models. The residue range of the peptides are indicated.

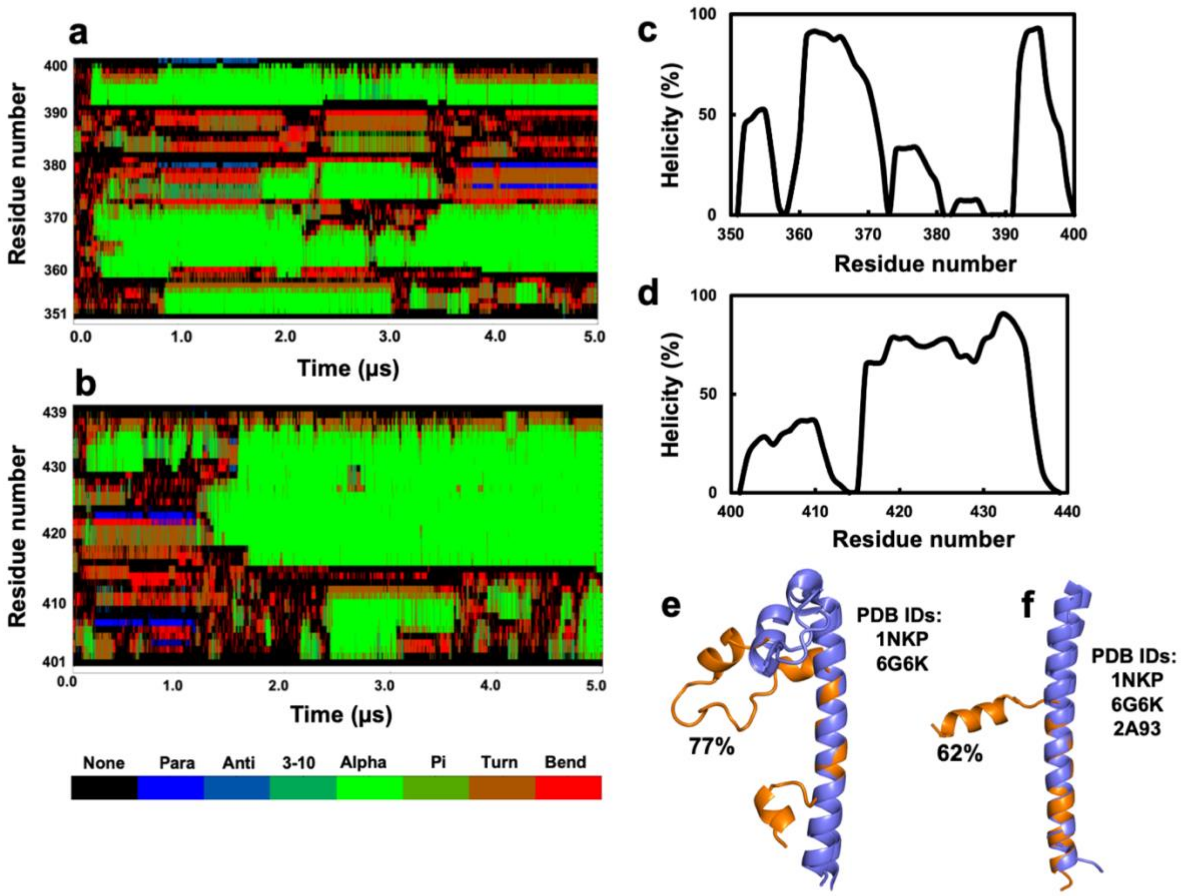

Supplementary Figure 2. Secondary structures of c-MYC peptides corresponding to bHLHZip domain. (a-b) Secondary structure evolution of c-MYC peptides (Residues 351-400 and 401- 439) as a function of the simulation time analysed using the dictionary of protein secondary prediction (DSSP) algorithm[1] (Para: Parallel beta sheet, Anti: Anti-parallel beta sheet, 3-10: 3_{10} helix, Alpha: Alpha Helix, Pi: Pi Helix). **(c-d)** Residue-wise percentage helicity of the two peptides computed using "secstruct" command from the cpptraj module of AMBER 18[2]. The reported helicity is the sum of the "3_{10}" and "alpha" helices of the individual residues. **(e-f)** Representative structures from the most populated clusters of the two c-MYC peptides (orange) superimposed with the structurally equivalent segments from the experimentally resolved bHLH and Zip domains of c-MYC (blue with PDB codes). The percentage of structures in both the clusters are indicated. Structure-based clustering of the MD generated conformations of the two peptides was performed using average-linkage algorithm[3] with pairwise Root Mean Square Deviation (RMSD) as a distance matrix.

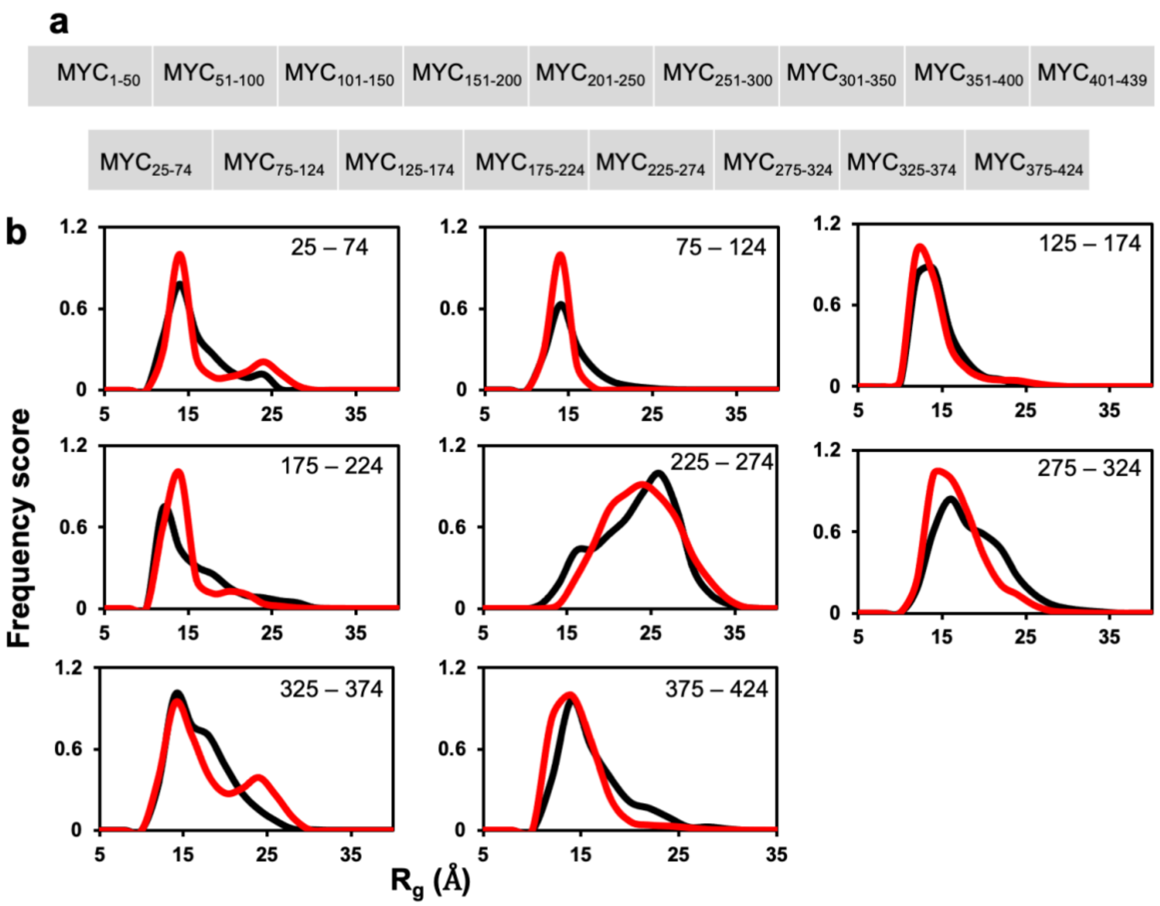

Supplementary Figure 3. Design and conformational sampling of c-MYC peptide derivatives. (a) Representation of the seventeen peptide derivatives of c-MYC analyzed. The nomenclature and residue range of each derivative are indicated. **(b)** Normalized frequency distribution of R_g computed from the ensemble of structures generated from explicit solvent MD simulations in water (black) and water + benzene (red) for the eight (MYC_{25-74} to $MYC_{375-424}$) c-MYC derived peptides whose residue range are indicated.

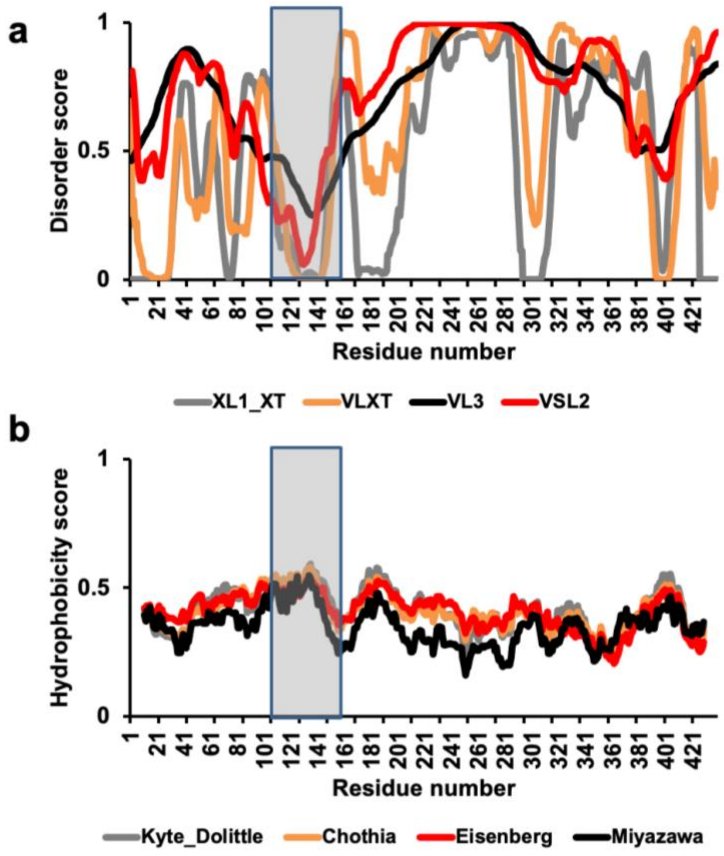

Supplementary Figure 4. Disorder and hydrophobicity. (a) Sequence-based prediction of disorder in c-MYC using four different algorithms as indicated through the Predictor of Natural Disorder Regions (PONDR) server. **(b)** Sequence-based prediction of hydrophobicity in c-MYC computed using four different hydrophobicity scale with a window size of 21 through the protscale analysis tool available on the SIB Expert Protein Analysis System (ExPASy) portal. The region highlighted with the rectangular box in both the plots corresponds to the coreMYC (residues 101-150) region of c-MYC.

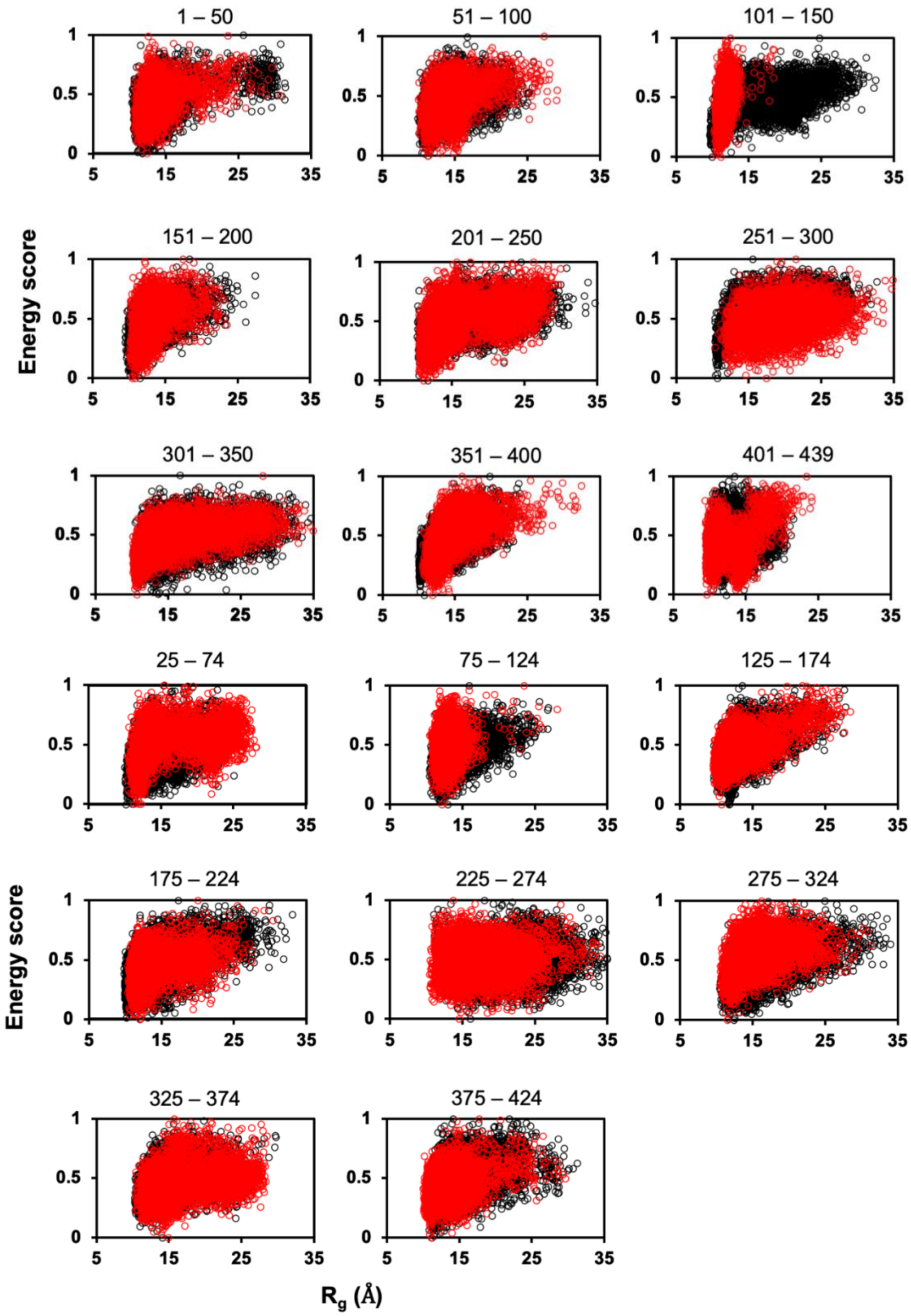

Supplementary Figure 5. Conformational sampling of c-MYC peptide derivatives. 2D scatter plots generated as a function of the radius of gyration (R_g) and the enthalpic energy (normalized in the scale from 0 to 1) for the ensemble of structures generated from MD simulations of the seventeen c-MYC peptide derivatives in water (black) and water + benzene (red) solvents. The residue range of each peptide is indicated above the respective plots.

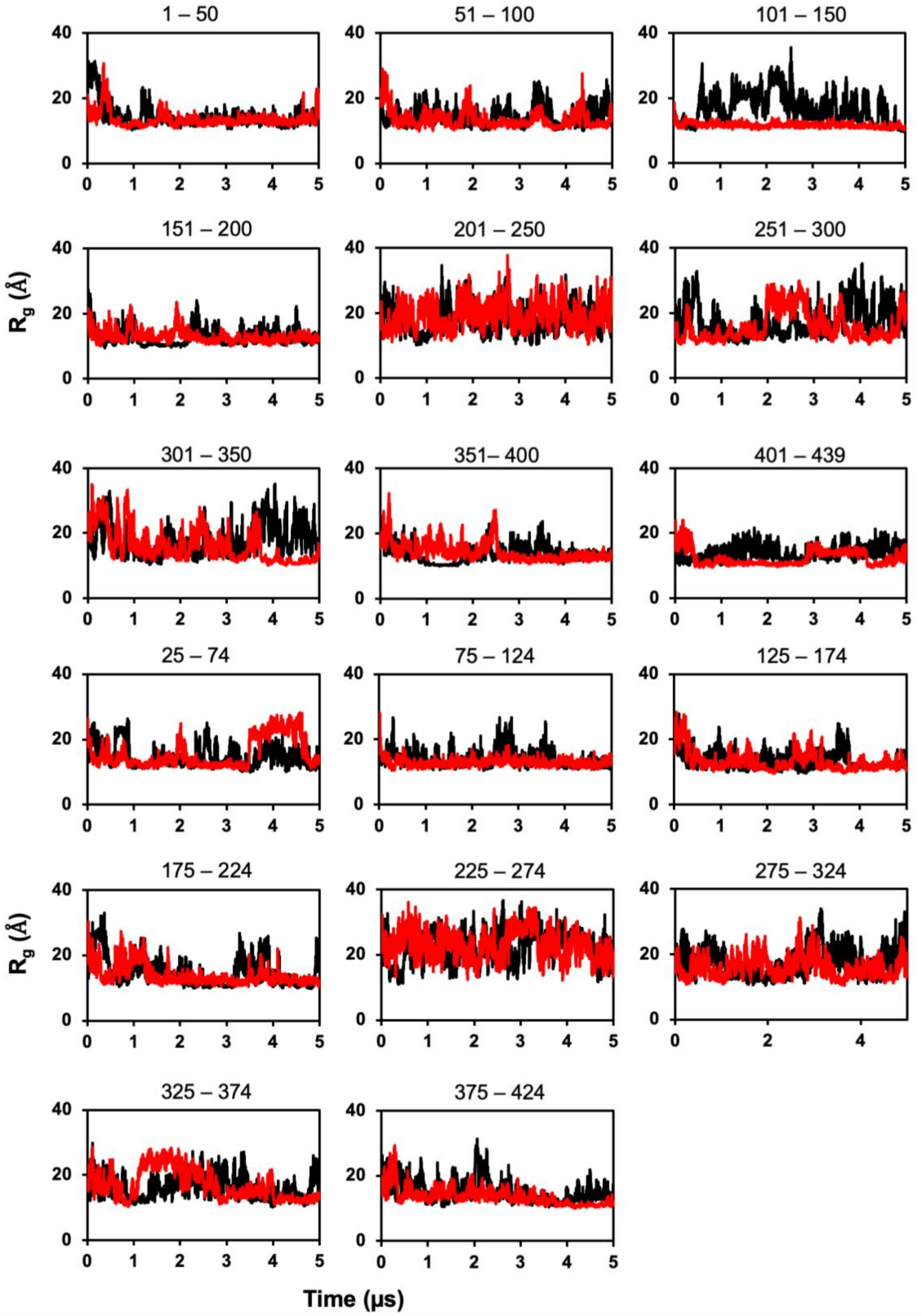

Supplementary Figure 6. Conformational fluctuation of c-MYC peptide derivatives. Radius of gyration (R_g) as a function of the simulation time of the seventeen c-MYC peptide derivatives in water (black) and water + benzene (red) solvents. The residue range of each peptide is indicated above the respective plots.

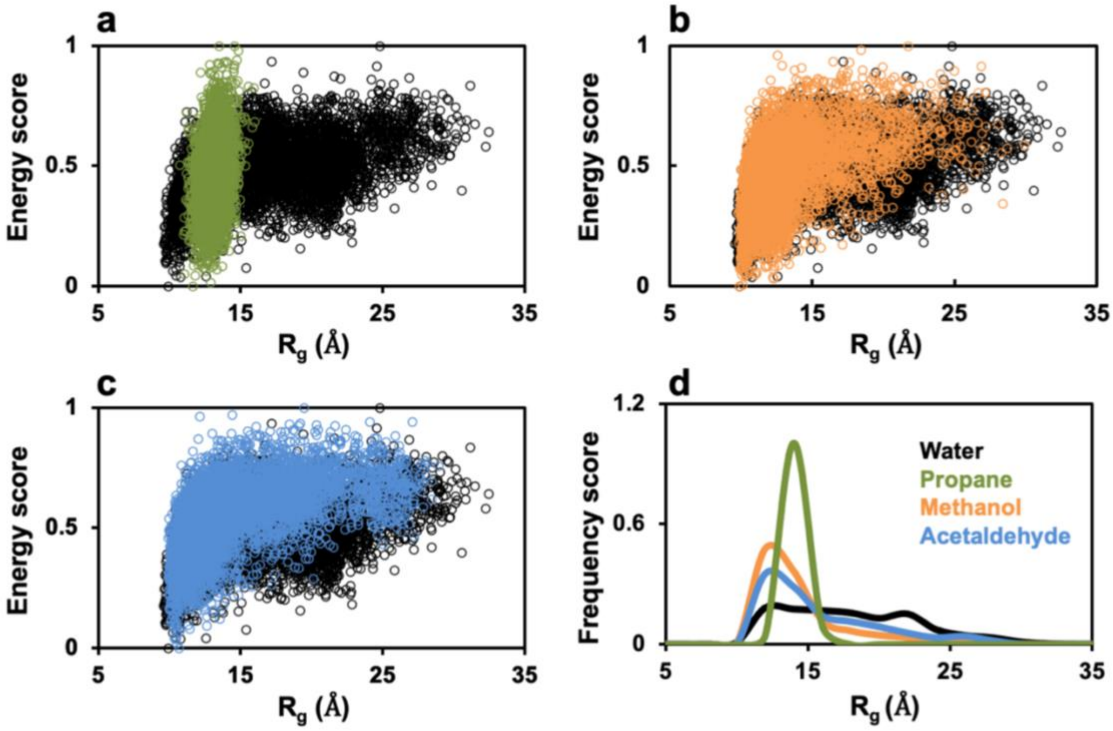

Supplementary Figure 7. Mixed-solvent simulations of coreMYC. (a-c) R_g-E (energy normalized in the scale from 0 to 1) distribution plots of coreMYC in mixed-solvent simulations of water with **(a)** propane (green), **(b)** methanol (orange), and **(c)** acetaldehyde (blue). R_g-E distribution of coreMYC in water (black) is shown for reference in all the three plots. **(d)** Normalized frequency distribution of R_g computed from the ensemble of structures generated from explicit solvent simulations in pure water and mixed-solvent simulations of water with propane, methanol, or acetaldehyde.

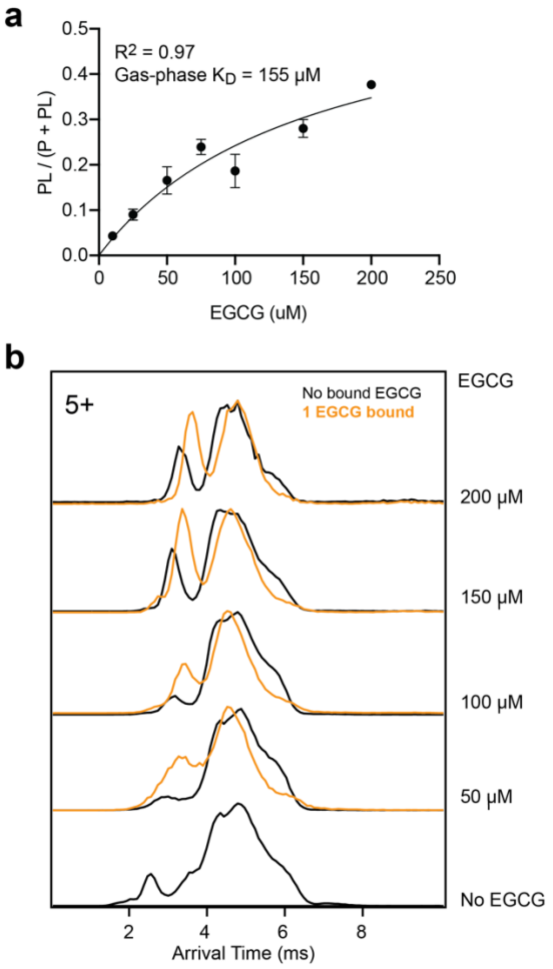

Supplementary Figure 8. Binding of EGCG to coreMYC in nMS and IM-MS. (**a**) nMS binding curve coreMYC and EGCG. 5 µM coreMYC was titrated with 0-200 µM EGCG and the mole fraction of the 1:1 complex plotted as a function of EGCG concentration. Error bars indicate the standard deviation of n = 3 independent repeats. (**b**) Arrival time distributions of apo coreMYC (black) and the 1:1 complex with EGCG (orange) for the 5+ charge state from IM-MS show an increase in compaction for the complex, as well as a minor increase in compact apo-coreMYC due to in-source dissociation of previously bound EGCG.

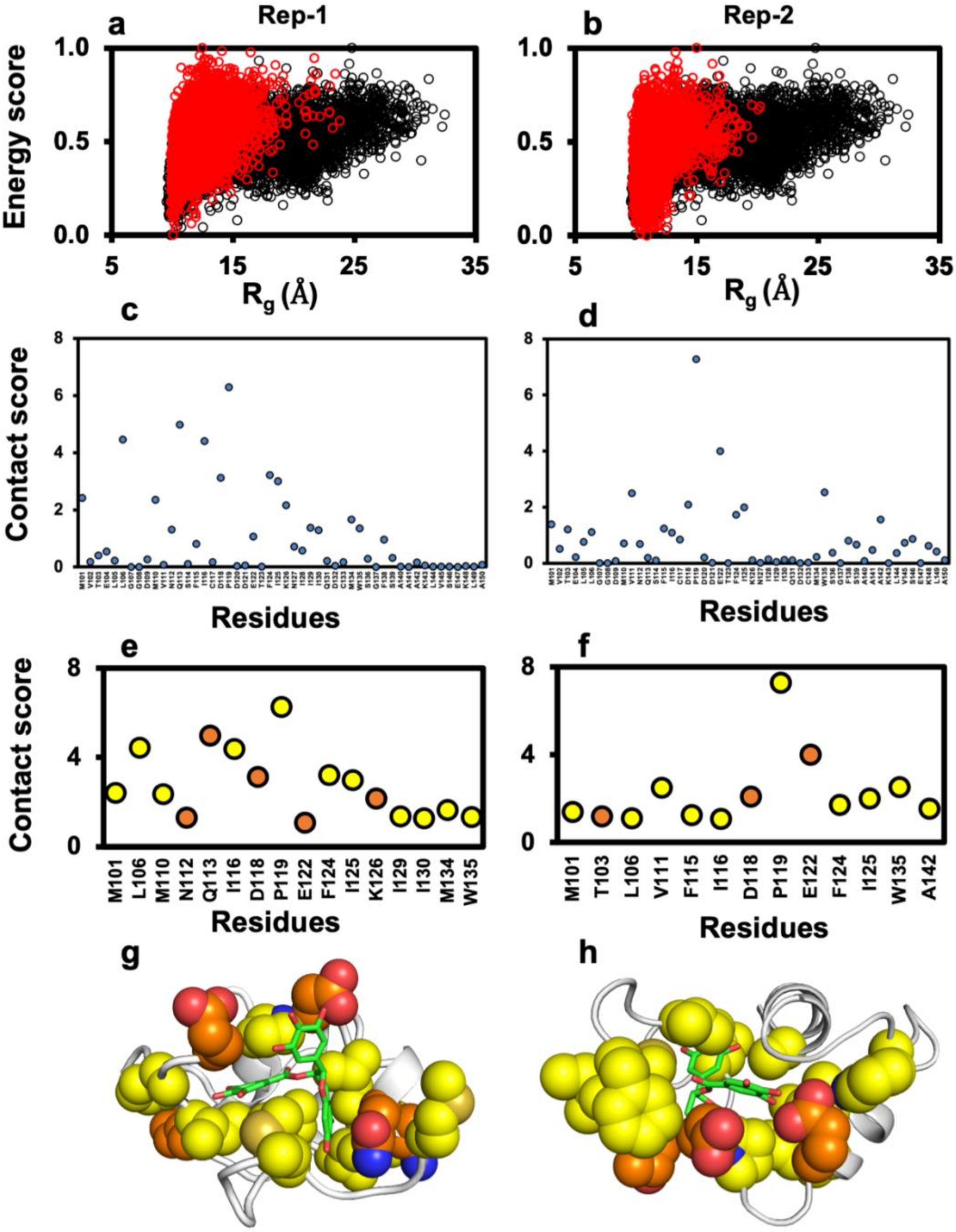

Supplementary Figure 9. Characterization of the interaction between coreMYC and EGCG. (a-b) R_g-E (energy normalized in the scale from 0 to 1) distribution plots of coreMYC generated from two independent MD simulations (Rep-1 and Rep-2) of the peptide in the presence of EGCG (red). R_g-E distribution of coreMYC in water (black) is shown for reference. **(c-d)** Residue contact map of coreMYC with EGCG from two independent MD simulations. **(e-f)** Subset of the residue contact map with contact score > 1. Dots corresponding to hydrophobic or hydrophilic residues are coloured in yellow and orange, respectively. **(g-h)** Representative structures from MD simulations of coreMYC bound to EGCG. The residues with contact score > 1 are shown in spheres and the EGCG is depicted in stick representation (green). Side-chain carbon atoms of hydrophobic residues are shown in yellow and hydrophilic residues in orange. Oxygen and nitrogen atoms are shown in red and blue, respectively.

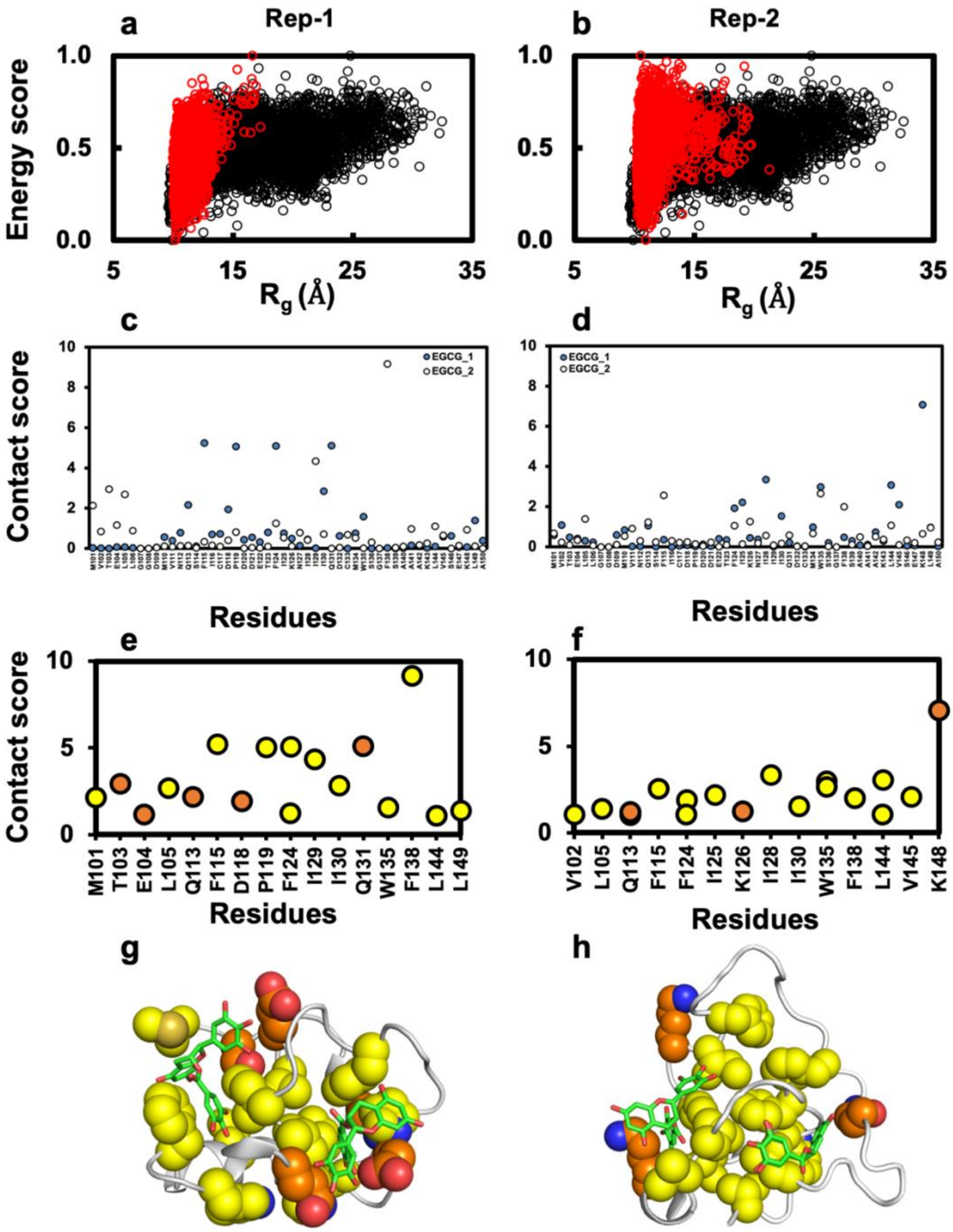

Supplementary Figure 10. Characterization of the interaction between coreMYC and two EGCG. (a-b) R_g-E (energy normalized in the scale from 0 to 1) distribution plots of coreMYC generated from two independent MD simulations (Rep-1 and Rep-2) of the peptide in the presence of two EGCG molecules (red). R_g-E distribution of coreMYC in water (black) is shown for reference. **(c-d)** Residue contact map of coreMYC with EGCG molecules from two independent MD simulations. **(e-f)** Subset of the residue contact map with contact score > 1. Dots corresponding to hydrophobic or hydrophilic residues are coloured in yellow and orange, respectively. **(g-h)** Representative structures from MD simulations of coreMYC bound to EGCG. The residues with contact score > 1 are shown in spheres and the EGCG is depicted in stick representation (green). Side-chain carbon atoms of hydrophobic residues are shown in yellow and hydrophilic residues in orange. Oxygen and nitrogen atoms are presented in red and blue, respectively.

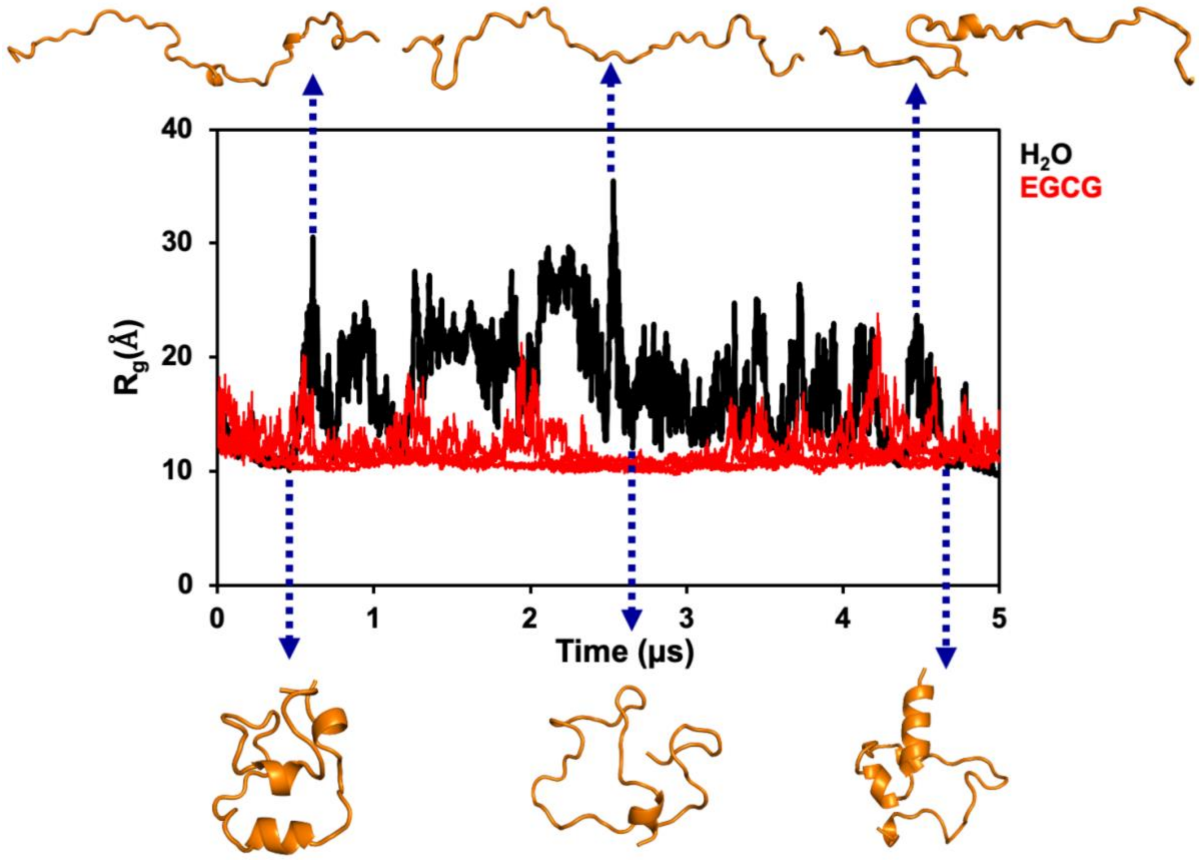

Supplementary Figure 11. Conformational fluctuation of coreMYC. Radius of gyration (R_g) of coreMYC as a function of the simulation time in water (black) and in the presence of one or two EGCG molecules (red). Representative structures of the extended and compact states of coreMYC are indicated.

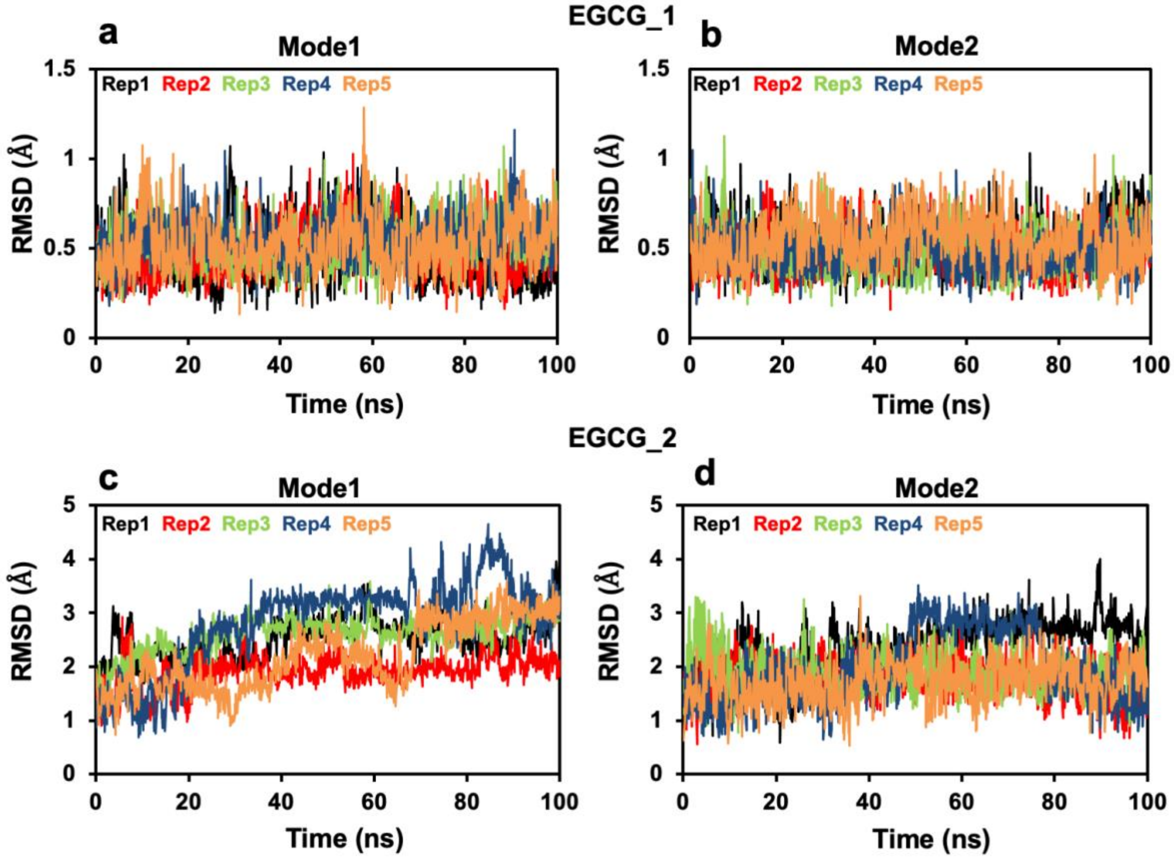

Supplementary Figure 12. Stability of EGCG bound to coreMYC. (a-d) Time-dependent Root mean square deviation (RMSD) of EGCG for five independent simulations (Rep1 to Rep5) of systems with one (EGCG_1) or two (EGCG_2) EGCG molecules bound to coreMYC. The representative binding modes (termed Mode1 and Mode2) of coreMYC: EGCG complex (as shown in Supplementary Figures 9g-h and 10g-h) were used as starting structures for these simulations. The RMSD was measured with reference to the starting structure for the respective systems.

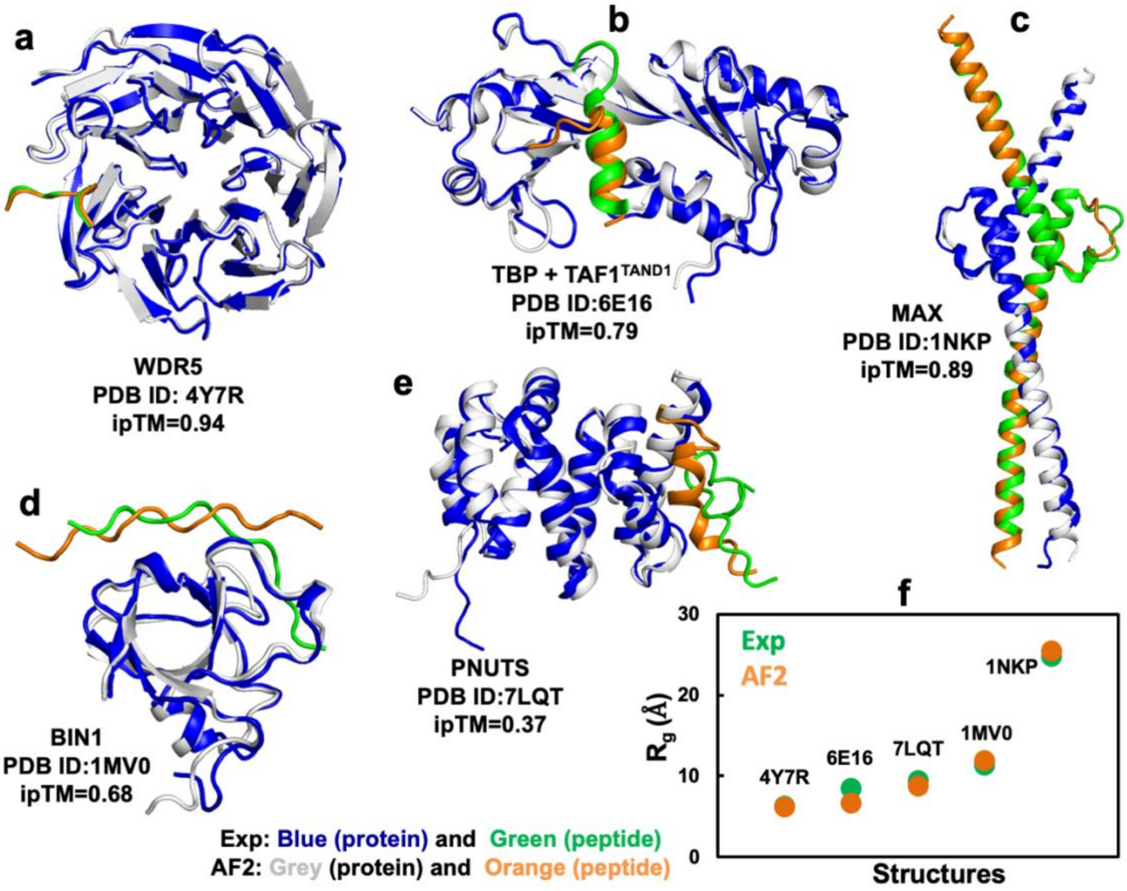

Supplementary Figure 13. Structural validation of AF2 predictions of c-MYC complexes. (a-e) Superimposition of experimentally resolved (blue and green) c-MYC complexes with the top-ranked AF2 models (grey and orange). The binding partners of c-MYC, PDB code of the experimental structures, and the ipTM scores from AF2 of the different complexes are indicated. **(f)** Radius of gyration (R_g) of c-MYC in the complexes computed from experimental (green) and AF2 (orange) structures.

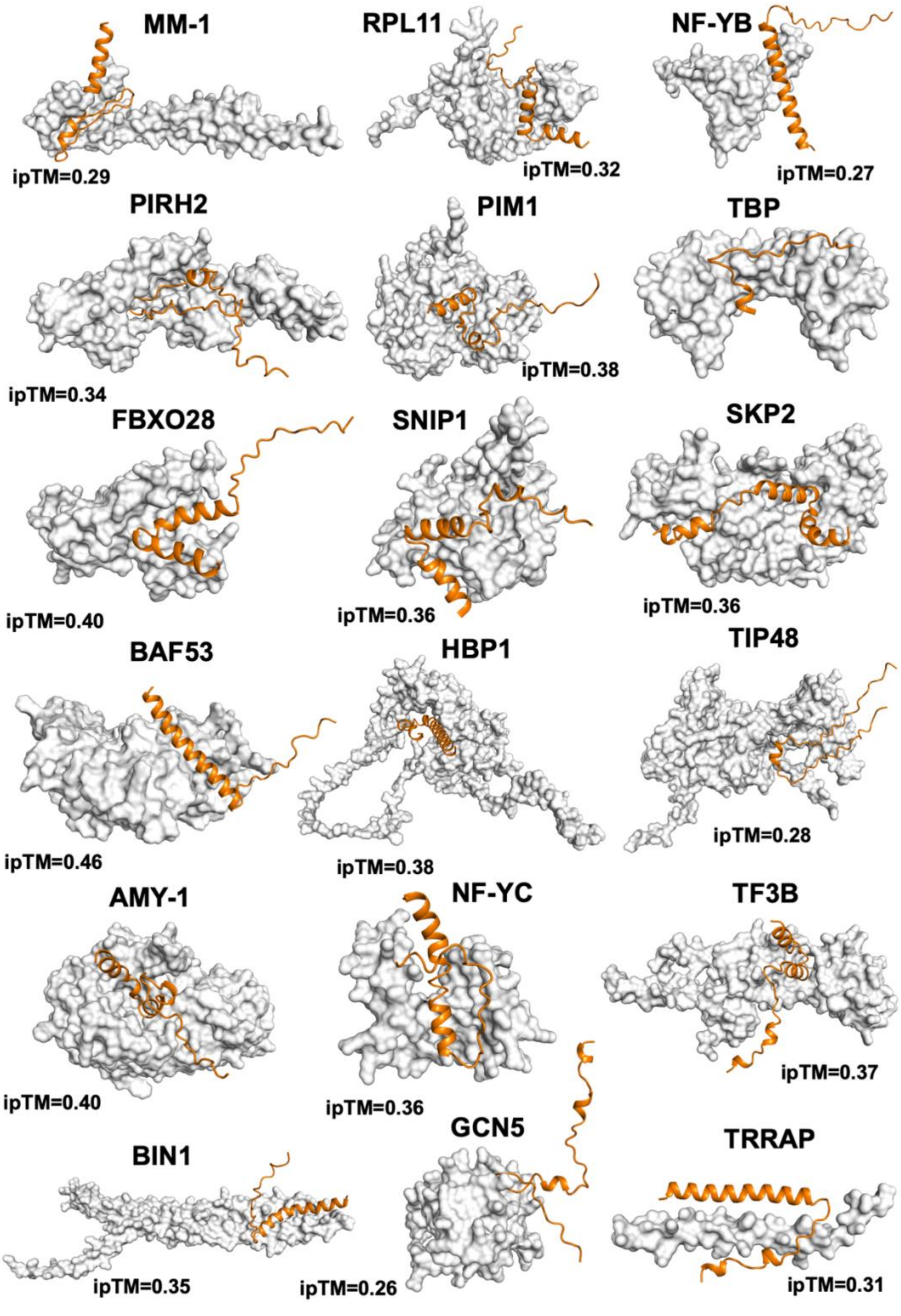

Supplementary Figure 14. Structure prediction of coreMYC:protein complexes. Representative structures of coreMYC (in orange) modelled in complex with its binding partners rendered in grey surface representation as indicated. All models were generated using AF2 except for TBP, where the complex was modelled for the first 24 residues of coreMYC and the core domain of human TBP (Residues 159-337: uniport ID: P20226) guided by the structural and biochemical data obtained from Wei *et al*[4]. For TRRAP, the AF2 model was generated for residues 2038-2087.

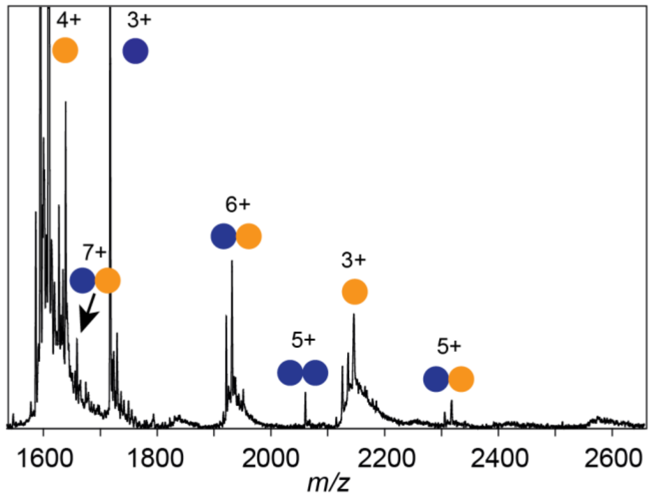

Supplementary Figure 15. Zoomed native mass spectrum of coreMYC and TRRAP. A total of three consecutive charge states can be detected for the coreMYC-TRRAP complex, as well as a minor population of TRRAP dimers. TRRAP and coreMYC are indicated by blue and orange circles, respectively.

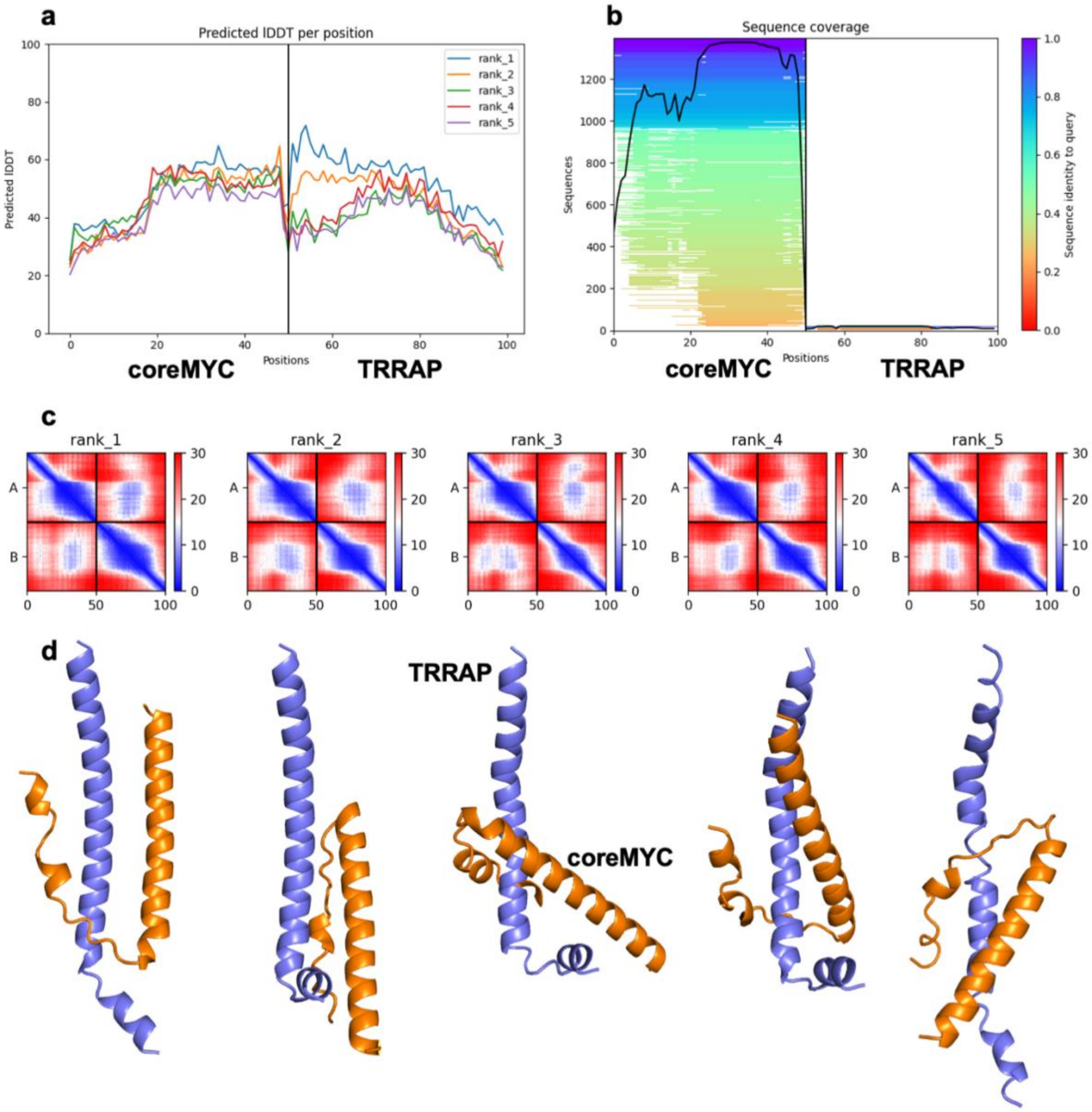

Supplementary Figure 16. AF2 generated models of the coreMYC:TRRAP$_{2038\text{-}2087}$ complex. Output from AF2 model building showing the **(a)** sequence coverage, **(b)** IDDT score, and the **(c)** residue contact map for the top five predicted models. **(d)** Representative structures of the modelled complexes shown in cartoon representation with coreMYC in orange and TRRAP$_{2038\text{-}2087}$ in blue.

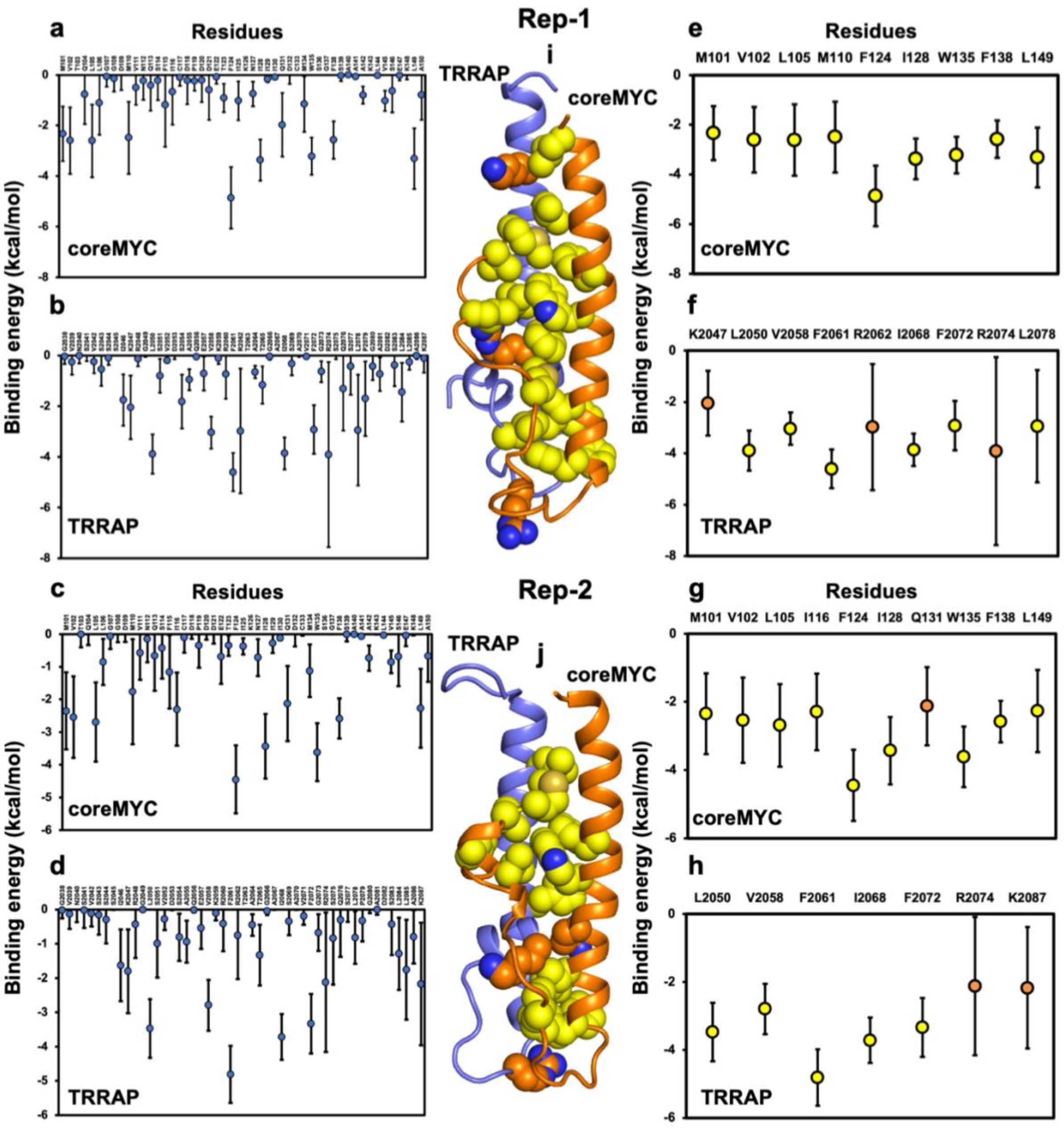

Supplementary Figure 17. Modelling coreMYC:TRRAP$_{2038-2087}$ complex. (a-d) Residue-wise binding energy contribution from the ensemble of structures generated from two independent MD simulations (Rep-1 and Rep-2) of the coreMYC:TRRAP$_{2038-2087}$ complex. Data are represented as Average±S.D. computed with n=1000 simulated structures. These simulations were started from the highest ranked AF2 generated model. **(e-h)** Subset of the residues with binding energy contribution ≤ -2 kcal/mol. Data are represented as Average±S.D. computed with n=1000 simulated structures. The hydrophobic or hydrophilic residues are shown in yellow and orange dots, respectively. **(i-j)** Representative structures of coreMYC:TRRAP$_{2038-2087}$ complex from the two independent MD simulations. coreMYC (orange) and TRRAP$_{2038-2087}$ (blue) are shown in cartoon representations and the residues with binding energy contribution ≤ -2 kcal/mol are shown in sphere. Side-chain carbon atoms of hydrophobic residues are shown in yellow and hydrophilic residues in orange. Nitrogen atoms are shown in dark blue.

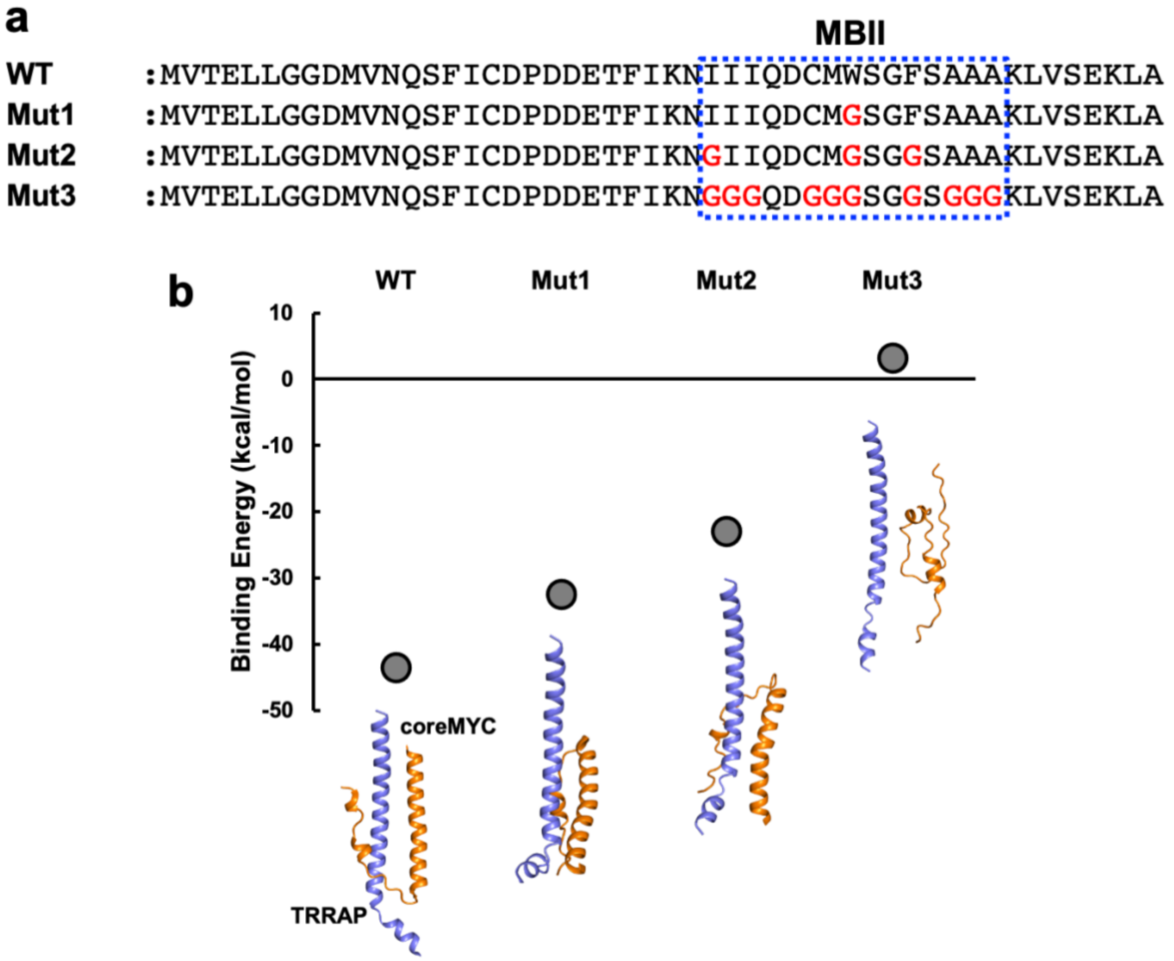

Supplementary Figure 18. ***In silico*** **generated glycine mutant derivatives of coreMYC. (a)** Amino acid sequences of wildtype (WT) coreMYC and the three glycine mutant derivatives (Mut1, Mut2, and Mut3). The MBII motif is shown in the blue box and the glycine (G) mutated residues are highlighted in red. **(b)** Binding energy of coreMYC and the three mutants with $TRRAP_{2038-2087}$ computed using MM/GBSA method[5] for the highest ranked AF2 generated models after energy minimization. Cartoon representation of the models are shown with coreMYC and its mutants in orange and $TRRAP_{2038-2087}$ in blue.

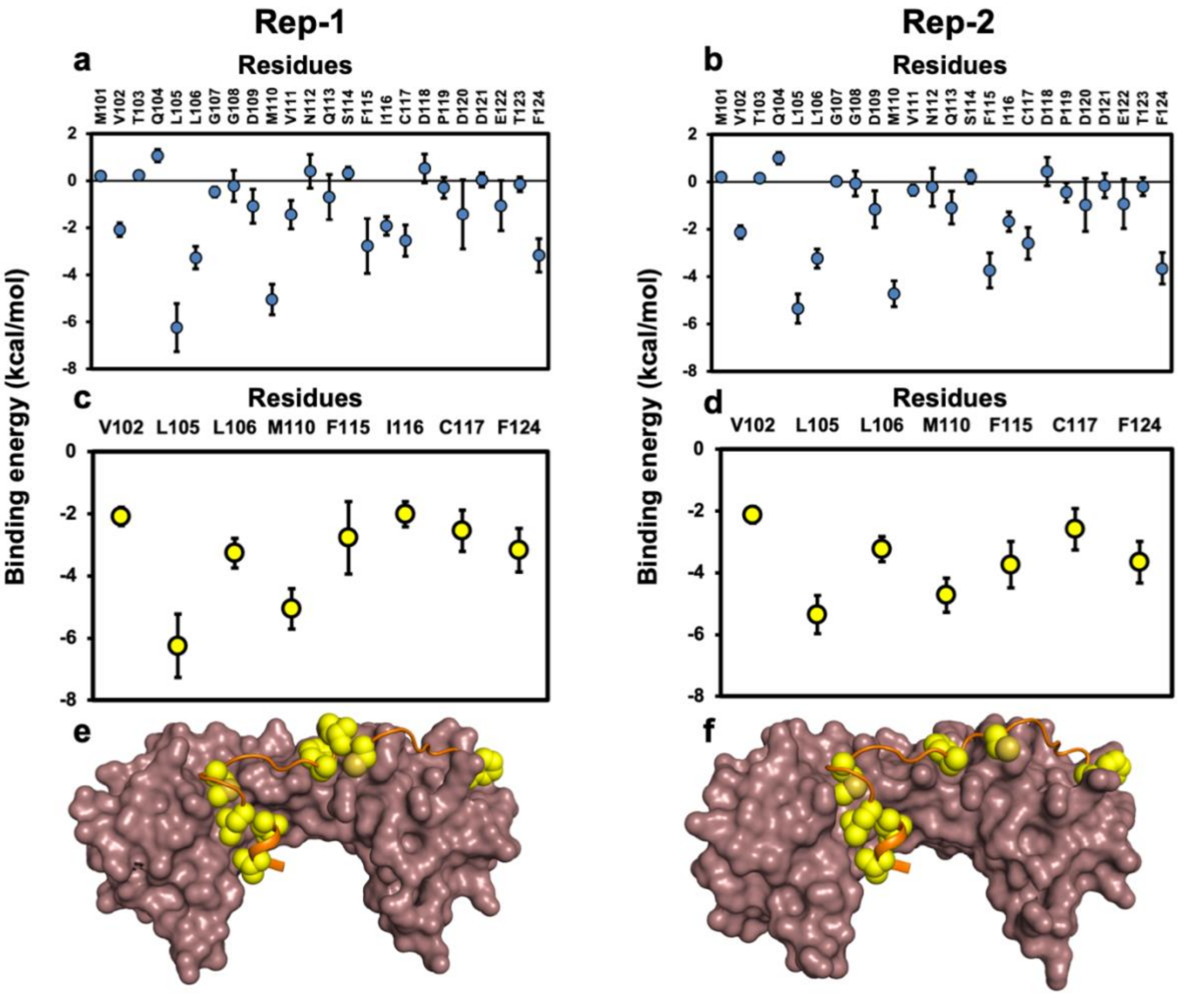

Supplementary Figure 19. Modelling the coreMYC:TBP complex. (a-b) Residue-wise binding energy contribution from the ensemble of structures generated from two independent MD simulations (Rep-1 and Rep-2) of the first 24 amino acids of coreMYC (residues 101-124) in complex with the core domain of human TBP (residues 159-337: uniport ID: P20226). Data are represented as Average±S.D. computed with n=1000 simulated structures. **(c-d)** Subset of the residues with binding energy contribution ≤ -2 kcal/mol. Data are represented as Average±S.D. computed with n=1000 simulated structures. **(e-f)** Representative structures of coreMYC:TBP complex from the two independent MD simulations. coreMYC (orange) and TBP (brown) are shown in cartoon and surface representations, respectively. The residues from coreMYC with binding energy contribution ≤ -2 kcal/mol are depicted in yellow spheres.

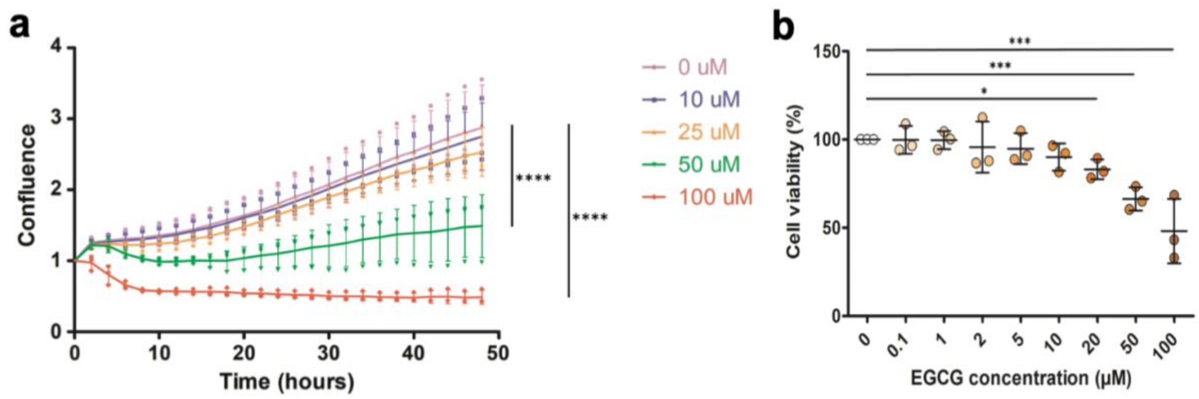

Supplementary Figure 20. Live-cell imaging and viability of EGCG-treated SH-SY5Y cells. (a) Cell viability represented by cell confluence as a function of EGCG concentrations as indicated. Cells were treated with EGCG and imaged continuously over a period of 48 hours with imaging over a repeated schedule with 2-hour imaging intervals. The live-cell imaging experiment was performed in three independent biological experiments with four replicates per repeat. Data is represented as mean±S.D. for each individual EGCG concentration. **(b)** Cell survival as a function of EGCG concentration as indicated. The cells were incubated with EGCG for 24 hours and cell viability was measured using WST1 colorimetric assay. The assay was performed in three independent biological experiments with four replicates per repeat. Asterisks indicate *p*-values of * 0.05 and *** 0.005, respectively.

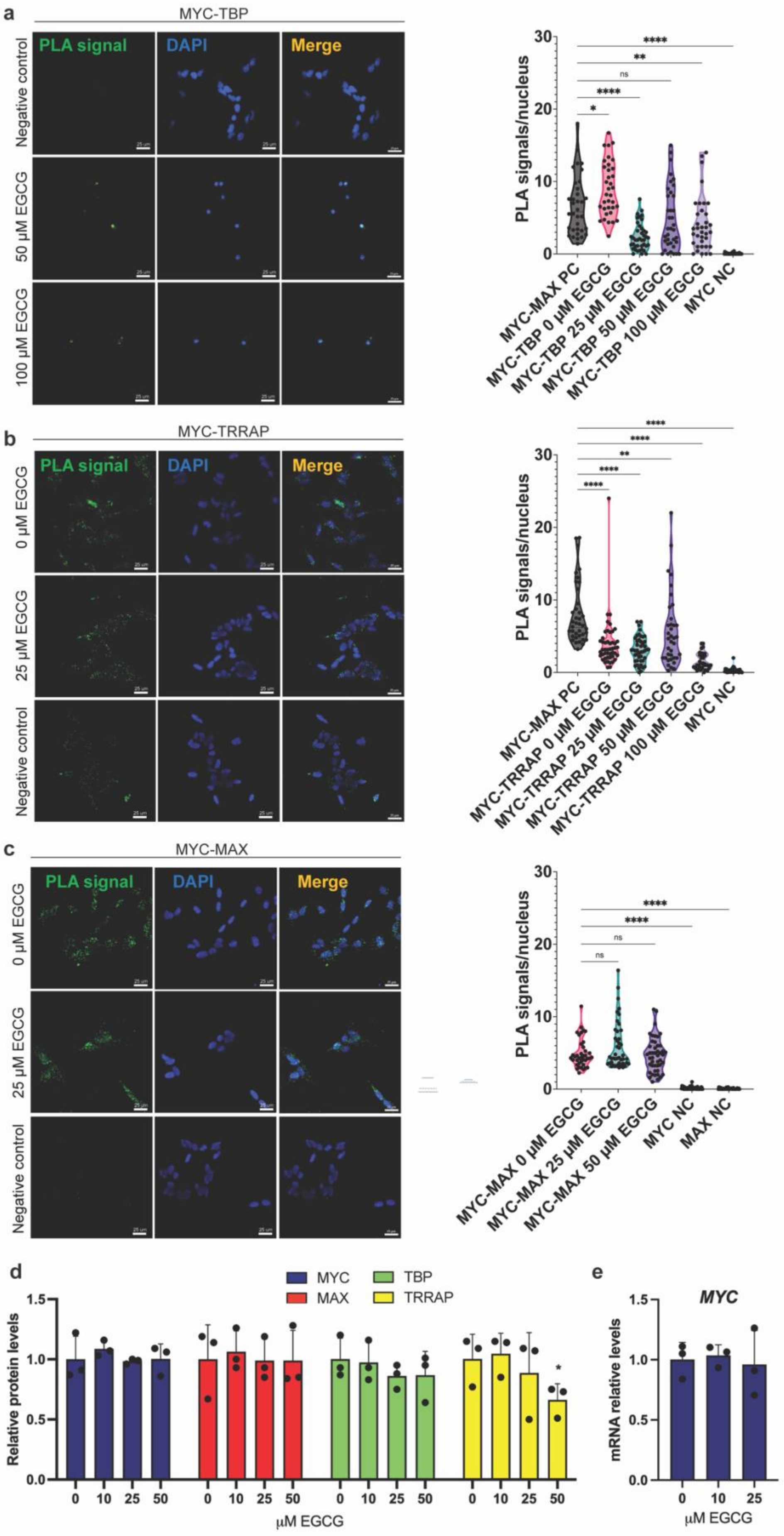
a
MYC-TBP
PLA signal
DAPI
Merge
Negative control
50 µM EGCG
100 µM EGCG
PLA signals/nucleus
MYC-MAX PC
MYC-TBP 0 µM EGCG
MYC-TBP 25 µM EGCG
MYC-TBP 50 µM EGCG
MYC-TBP 100 µM EGCG
MYC NC
b
MYC-TRRAP
0 µM EGCG
25 µM EGCG
MYC-TRRAP 0 µM EGCG
MYC-TRRAP 25 µM EGCG
MYC-TRRAP 50 µM EGCG
MYC-TRRAP 100 µM EGCG
c
MYC-MAX
MYC-MAX 0 µM EGCG
MYC-MAX 25 µM EGCG
MYC-MAX 50 µM EGCG
MAX NC
d
MYC
MAX
TBP
TRRAP
Relative protein levels
µM EGCG
e
MYC
mRNA relative levels
µM EGCG

Supplementary Figure 21. EGCG inhibits interactions between c-MYC (MYC) and TBP and TRRAP, but not MAX, in cancer cells. (a) Interaction of c-MYC and TBP. **Left panel:** Representative images of PLA signals for c-MYC in interaction with TBP in SH-SY5Y cells (n = 3 biologically independent experiments). Top row: Negative control showing no PLA signal (green) for c-MYC:MAX as only c-MYC antibody was used. Middle row: PLA for the cMYC:TBP interaction (green) in SH-SY5Y cells treated with 50 µM EGCG. Bottom row: PLA for the c-MYC:TBP complex (green) in cells treated with 100 µM EGCG. Nuclei were stained with DAPI (blue). Yellow indicates the merge between the respective PLA signal with DAPI. Scale bar is 25 µm. **Right panel:** Quantification of the nuclear c-MYC:TBP PLA signal in cells treated with the indicated concentrations of EGCG and for the negative control. Experiments were performed three times. Asterisks indicate a *p*-value of **** *p*=<0.0001, n.s., non-significant. **(b)** Interaction of c-MYC and TRRAP, as for (a) but with 0 and 25 µM EGCG in the left panel as indicated. **(c)** Interaction of c-MYC and MAX, as for (a) but with 0 and 25 µM EGCG in the left panel as indicated. **(d)** Western blot quantification of the expression levels of MYC, MAX, TBP; and TRRAP in response to increasing concentrations of EGCG during 24 hrs. Error bars indicate standard deviation of n = 3 biologically independent experiments. **(e)** RT-qPCR of *MYC* in SH-SY5Y cells treated for 24 hrs with the indicated concentrations of EGCG. Data was normalized to the housekeeping gene (*β-2-microglobulin, B2M*) and represented relative to the control (0 µM EGCG). Mean ±S.D. of n = 3 biologically independent experiments (with three technical replicates per biological experiment), not significant between conditions. Statistical analysis: two-tailed unpaired *t*-test.

Supplementary Table 1. List of selected proteins for AF2 complex modelling with coreMYC. The information on the proteins and their region of interaction with c-MYC were taken from Tu *et al*,[6]. The residue range defined for MBII is 128-143.

Sl. No.	Protein	Uniprot ID	Residue Length	Region of interaction within c-MYC
1	MM-1	Q99471	154	amino-terminus (104-166)
2	RPL11	P62913	178	MBII
3	NF-YB	P25208	207	amino-terminus (MBI and/or MBII)
4	PIRH2	Q96PM5	261	amino-terminus (120-160)
5	PIM1	P11309	313	MBII
6	TBP	P20226	339	amino-terminus (1-143)
7	FBXO28	Q9NVF7	368	MBII
8	SNIP1	Q8TAD8	396	amino-terminus (2-147)
9	SKP2	Q13309	424	MBII
10	BAF53	O96019	429	MBII
11	NF-YC	Q13952	458	amino-terminus (MBI and/or MBII)
12	TIP48	Q9Y230	463	MBII
13	AMY-1	P0DTE8	511	amino-terminus (104-143, including MBII)
14	HBP1	O60381	514	MBII
15	BIN1	O00499	593	MBII
16	TF3B	Q92994	677	amino-terminus (1-262)
17	GCN5	Q92830	837	MBII
18	TRRAP	Q9Y4A5	3859	amino-terminus [24-31, 38-48, MBII (129-145)]

Supplementary Table 2. Details on the volume of the simulation box and the number of benzene molecules added for the mixed-solvent simulations of each peptide. For $MYC_{101-150}$ (coreMYC), the figures also corresponds to the number of propane, methanol and acetaldehyde added in the additional mixed-solvent simulations of the peptide.

Peptides	Residue range	Volume of the simulation box (Å^3)	Number of benzene molecules
MYC_{1-50}	1-50	233102.761	56
MYC_{51-100}	51-100	362523.154	87
$MYC_{101-150}$	101-150	235165.072	60
$MYC_{151-200}$	151-200	331881.146	80
$MYC_{201-250}$	201-250	362244.787	87
$MYC_{251-300}$	251-300	263933.383	67
$MYC_{301-350}$	301-350	294735.611	71
$MYC_{401-450}$	351-400	284109.543	68
$MYC_{401-439}$	401-439	221051.228	53
MYC_{25-74}	25-74	223673.053	54
MYC_{75-124}	75-124	223322.285	54
$MYC_{125-174}$	125-174	228978.732	55
$MYC_{175-224}$	175-224	235165.072	56
$MYC_{225-274}$	225-274	223760.802	54
$MYC_{275-324}$	275-324	253321.411	61
$MYC_{325-374}$	325-374	244651.949	59
$MYC_{375-424}$	375-424	250756.230	60

Supplementary Table 3. Forward (F) and Reverse (R) primers used for real-time quantitative polymerase chain reaction.

**Gene**	**Sequence (5'-3')**
β-2-microglobulin (B2M)	F: TGCTGTCTCCATGTTTGATGTATC<br>R: TCTCTGCTCCCCACCTCTAAGT
c-MYC	F: CATCAGCACAACTACGCAGC<br>R: CGTTGTGTGTTCGCCTCTTG

Supplementary Note

Assessment of force-field and water model for MD simulations

A major concern with atomistic simulations of IDPs employing currently available protein force-fields is their tendency to favour overly compact structures[7,8]. In this regard, we carried out an independent assessment of three different AMBER-based protein force-fields (ff14SB, ff14IDPSFF, and ff19SB) with two water models (TIP3P and OPC) to sample the conformational states of nine non-overlapping c-MYC peptide derivatives (Supplementary Figure 1). We found that ff14SB[9] with the three-point rigid TIP3P[10] water model sampled only the compact states for all the nine peptides. The combination of ff14IDPSFF[11] (a force-field tailored for IDPs) and TIP3P relatively improved the sampling property of the peptides. Significantly, the most recently developed AMBER force-field ff19SB[12], which has been parametrized with four point rigid OPC[13] water model was able to sample the highest structural diversity with exploration of extended states across the different peptides. Based on this evaluation, we performed our simulations with the ff19SB force-field and OPC water model.

The peptides spanning residues 351-400 and 401-439 corresponds together to the structurally well-characterized bHLHZip domain of c-MYC[14-16] .The secondary structure evolution during MD simulations of these two peptides initiated from an extended conformation showed significant presence of helical elements (Supplementary Figures 2a-b). In $MYC_{351-400}$, residues 361-370 and 392-396 adopt a helical backbone geometry in more than 50% of the structures (Supplementary Figure 2c), while for $MYC_{401-439}$, a single pre-dominant helical segment is observed between residues 416-435 (Supplementary Figure 2d). The formation of the Helix-Loop-Helix (HLH) topology in $MYC_{351-400}$ and the helical Leucine Zipper (Zip) in $MYC_{401-439}$ (Supplementary Figures 2e-f) indicate that the carboxy-terminal region of c-MYC adopts a helical geometry in solution, and is pre-disposed towards the MAX bound conformations observed in experimentally resolved structures[14-16] .

Supplementary References

1. W. Kabsch, C. Sander, Dictionary of protein secondary structure: pattern recognition of hydrogen-bonded and geometrical features. *Biopolymers*. 22, 2577-637 (1983).
2. D.A. Case et al., AMBER 18. *University of California, San Francisco* (2018).
3. J. Shao, S.W. Tanner, N. Thompson, T.E. Cheatham, Clustering Molecular Dynamics Trajectories: 1. Characterizing the Performance of Different Clustering Algorithms. *J Chem Theory Comput*. 3, 2312-34 (2007).
4. Y. Wei et al., Multiple direct interactions of TBP with the MYC oncoprotein. *Nat Struct Mol Biol*. 26, 1035-1043 (2019).
5. S. Genheden, U. Ryde, The MM/PBSA and MM/GBSA methods to estimate ligand-binding affinities. *Expert Opin Drug Discov*. 10, 449-461 (2015).
6. W.B. Tu et al., Myc and its interactors take shape. *Biochim Biophys Acta*. 1849, 469-83 (2015).
7. J. Huang, A.D. MacKerell Jr, Force field development and simulations of intrinsically disordered proteins. *Curr Opin Struct Biol*. 48, 40-48 (2018).
8. P.S. Shabane, S. Izadi, A.V. Onufriev, General Purpose Water Model Can Improve Atomistic Simulations of Intrinsically Disordered Proteins. *J Chem Theory Comput*. 15, 2620-2634 (2019).
9. J. A. Maier et. al., ff14SB: Improving the Accuracy of Protein Side Chain and Backbone Parameters from ff99SB. *J Chem Theory Comput*. 11, 3696-713 (2015).
10. W.L. Jorgensen, J. Chandrasekhar, J.D. Madura, R.W. Impey, M.L. Klein, Comparison of simple potential functions for simulating liquid water. *J Chem Phys*. 79, 926-935, (1983).
11. D. Song, R. Luo, H.F. Chen, The IDP-Specific Force Field ff14IDPSFF Improves the Conformer Sampling of Intrinsically Disordered Proteins. *J Chem Inf Model*. 57, 1166-1178 (2017).
12. C. Tian et. al., ff19SB: Amino-Acid-Specific Protein Backbone Parameters Trained against Quantum Mechanics Energy Surfaces in Solution. *J Chem Theory Comput*. 16, 528-552 (2020).
13. S. Izadi, R. Anandakrishnan, A.V. Onufriev, Building Water Models: A Different Approach. *J Phys Chem Lett*. 5, 3863-3871 (2014).
14. S. Sammak et. al., Crystal Structures and Nuclear Magnetic Resonance Studies of the Apo Form of the c-MYC:MAX bHLHZip Complex Reveal a Helical Basic Region in the Absence of DNA. *Biochemistry*. 58, 3144-3154 (2019).
15. S.K. Nair, S.K. Burley, X-ray structures of Myc-Max and Mad-Max recognizing DNA. Molecular bases of regulation by proto-oncogenic transcription factors. *Cell* 112, 193-205 (2003).
16. P. Lavigne et. al., Insights into the mechanism of heterodimerization from the 1H-NMR solution structure of the c-Myc-Max heterodimeric leucine zipper. *J Mol Biol*. 281, 165-81 (1998).
