# Supplementary material for: A druggable conformational switch in the c-MYC transactivation domain
Source: Nat Commun. 2024 Feb 29;15:1865. doi: 10.1038/s41467-024-45826-7 (PMC10904854; doi:10.1038/s41467-024-45826-7)
Supplement: Supplementary file 3 — Reporting Summary [file 41467_2024_45826_MOESM3_ESM.pdf]

nature portfolio

Corresponding author(s): Dilraj Lama, Michael Landreh and Marie Arsenian Henriksson

Last updated by author(s): Jan 30, 2024

Reporting Summary

Nature Portfolio wishes to improve the reproducibility of the work that we publish. This form provides structure for consistency and transparency in reporting. For further information on Nature Portfolio policies, see our Editorial Policies and the Editorial Policy Checklist.

Statistics

For all statistical analyses, confirm that the following items are present in the figure legend, table legend, main text, or Methods section.

n/a	Confirmed	
☐	☒	The exact sample size (*n*) for each experimental group/condition, given as a discrete number and unit of measurement
☐	☒	A statement on whether measurements were taken from distinct samples or whether the same sample was measured repeatedly
☐	☒	The statistical test(s) used AND whether they are one- or two-sided<br>*Only common tests should be described solely by name; describe more complex techniques in the Methods section.*
☐	☒	A description of all covariates tested
☒	☐	A description of any assumptions or corrections, such as tests of normality and adjustment for multiple comparisons
☐	☒	A full description of the statistical parameters including central tendency (e.g. means) or other basic estimates (e.g. regression coefficient) AND variation (e.g. standard deviation) or associated estimates of uncertainty (e.g. confidence intervals)
☐	☒	For null hypothesis testing, the test statistic (e.g. *F*, *t*, *r*) with confidence intervals, effect sizes, degrees of freedom and *P* value noted<br>*Give P values as exact values whenever suitable.*
☒	☐	For Bayesian analysis, information on the choice of priors and Markov chain Monte Carlo settings
☒	☐	For hierarchical and complex designs, identification of the appropriate level for tests and full reporting of outcomes
☒	☐	Estimates of effect sizes (e.g. Cohen's *d*, Pearson's *r*), indicating how they were calculated

Our web collection on statistics for biologists contains articles on many of the points above.

Software and code

Policy information about availability of computer code

Data collection	MassLynx V 4.1 (commercial, Waters, UK) for all MS data collection. All Molecular Dynamic (MD) Simulations were performed using AMBER 18 employing ff19SB force field parameters and OPC water model. Protein structure prediction using Alphafold2 (AF2) was done through the jupyter notebook for Colabfold (v 1.3.0). WST-1 cell viability data was recorded with SparkControl magellan version 1.2 (Tecan, Switzerland). IncuCyte viability data was collected using the IncuCyte software 2021C (Essen Bioscience Inc., MI, USA). Proximity ligation assay images were recorded using the NIS-Elements Advanced Research 5.02.03 software (Nikon, NY, USA). Western blots were developed in a ChemiDoc XRS+ System (Bio-Rad, Hercules, CA, USA). RT-qPCR was performed in a StepOnePlus Real-Time PCR system (Applied Biosystems, Waltham, MA, USA).
Data analysis	MassLynx V 4.1 (commercial, Waters, UK) for all MS data analysis. IM-MS data was analyzed via Pulsar v2.0 and theoretical CCS values from PDB files were computed with IMPACT. MD data analysis was done using the Ambertools suite of programs available in AMBER 18. Molecular visualization of MD trajectories and generated structures was done with VMD v 1.9.3. and PyMOL v 2.2.0. PLA positive signals were quantified using the NIS-Elements Advanced Research 5.02.03 software (Nikon, NY, USA). Western blot bands were quantified with ImageJ (NIH, Bethesda, MD, USA).<br>WST-1 cell viability, PLA data, WB quantification and RT-qPCR data analysis were performed using GraphPad Prism V 5.0.4 (GraphPad Software Inc., San Diego, CA, USA). IncuCyte viability data was analyzed using the IncuCyte software 2021C (Essen Bioscience Inc., MI, US) and GraphPad Prism V 5.0.4 (GraphPad Software Inc., San Diego, CA, USA).

For manuscripts utilizing custom algorithms or software that are central to the research but not yet described in published literature, software must be made available to editors and reviewers. We strongly encourage code deposition in a community repository (e.g. GitHub). See the Nature Portfolio guidelines for submitting code & software for further information.

Data

Policy information about availability of data

All manuscripts must include a data availability statement. This statement should provide the following information, where applicable:

- Accession codes, unique identifiers, or web links for publicly available datasets
- A description of any restrictions on data availability
- For clinical datasets or third party data, please ensure that the statement adheres to our policy

The molecular dynamics simulations trajectories reported in this study has been deposited in the Figshare repository (https://figshare.com/articles/journal_contribution/coreMYC/25020956). All data supporting the findings of this manuscript are available from the corresponding authors upon request. Source data are provided with this paper.

Human research participants

Policy information about studies involving human research participants and Sex and Gender in Research.

Reporting on sex and gender	Report on sex and gender is not relevant for our study as it does not involve human participants.
Population characteristics	Population characteristics is not relevant for our study as it does not involve human participants.
Recruitment	This information is not relevant for our study as it does not involve human participants.
Ethics oversight	This approval is not relevant for our study as it does not involve human participants.

Note that full information on the approval of the study protocol must also be provided in the manuscript.

Field-specific reporting

Please select the one below that is the best fit for your research. If you are not sure, read the appropriate sections before making your selection.

[x] Life sciences [ ] Behavioural & social sciences [ ] Ecological, evolutionary & environmental sciences

For a reference copy of the document with all sections, see nature.com/documents/nr-reporting-summary-flat.pdf

Life sciences study design

All studies must disclose on these points even when the disclosure is negative.

Sample size	No statistical methods were used to determine sample size. The chosen sample size for all conducted experiments being mass spectrometry, WST-1 viability assay, IncuCyte live-cell imaging viability assay, PLA, Western blots and RT-qPCR, was determined from similar experiments in the literature. For small scale experiments such as the ones we performed, the number of replicates is required to be at least 3 biologically independent replicates and 3 technical replicates. We complied with this as follows: for the WST-1 cell viability assay and the IncuCyte live-cell imaging viability assay, a total of four replicates per biologically independent replicate was performed. For the proximity ligation assay, a total of three independent experiments was performed. In each repeat, a minimum of 10 fields were captured for each condition during microscopic analysis to allow reliable PLA signal quantification, a minimum of 100 cells were randomly selected from each condition. The mass spectrometry experiments require a single repeat to acquire reliable data. For Western blot and RT-qPCR data, three independent experiments were performed. In the RT-qPCRs, three technical replicates per biologically independent replicate were analyzed.
Data exclusions	In the WST-1 cell viability assay, a variation of EGCG concentrations was used ranging from 0 to 100 uM. For one concentration, 30 uM, a volume measuring error occurred, resulting in outlying data. The error was confirmed and as a result the data points for 30 uM EGCG influence on SH-SY5Y cell viability were omitted.
Replication	Three repeats were performed per experiment to evaluate data replication and to reassure sufficient data were collected. All repeats revealed the same findings and confirmed one another. Standard deviations are displayed for the WST-1 cell viability assay to support repeatability of results, as well as for Western blot quantification and RT-qPCR data.
Randomization	Randomization was not relevant for our study, due to the small sample size. Experiments included were mass spectrometry, WST-1 cell viability assay, proximity ligation assay, Western blot and RT-qPCR. Neither of these typically are randomized prior to data collection or analysis.
Blinding	Blinding was not relevant for our study. Experiments included were mass spectrometry, cell viability assay, proximity ligation assay, Western blot and RT-qPCR. Blinding for data collection and/or analysis with regard to these experimental procedures is typically not performed. For mass spectrometry blinding is not required since the sample content is defined during analysis. Blinding is not relevant for our mass spectrometry experiments. The PLA, Western blots, RT-qPCR and IncuCyte viability assay were all prepared in small sample sizes and conducted by the same researchers who did sample preparation, data recording and data analysis. As per the literature for these

experiments, blinding was not needed for our experiments.

Reporting for specific materials, systems and methods

We require information from authors about some types of materials, experimental systems and methods used in many studies. Here, indicate whether each material, system or method listed is relevant to your study. If you are not sure if a list item applies to your research, read the appropriate section before selecting a response.

Materials & experimental systems

n/a	Involved in the study
☐	☒ Antibodies
☐	☒ Eukaryotic cell lines
☒	☐ Palaeontology and archaeology
☒	☐ Animals and other organisms
☒	☐ Clinical data
☒	☐ Dual use research of concern

Methods

n/a	Involved in the study
☒	☐ ChIP-seq
☒	☐ Flow cytometry
☒	☐ MRI-based neuroimaging

Antibodies

Antibodies used

For PLA assays, we used mouse monoclonal anti-c-MYC (9E10, sc-40, Lot# K2420, 1:200 dilution) and rabbit polyclonal anti-MAX (C-17, sc-197, Lot #J0809, 1:200 dilution), both from Santa Cruz Biotechnology (Dallas, TX, USA); rabbit polyclonal anti-TBP (ab63766, Lot GR3319877-6, 1:200 dilution) from Abcam (Cambridge, UK); rabbit polyclonal anti-TRRAP (SAB1300444, Lot #020M1982, 1:400 dilution) from Sigma-Aldrich (St. Louis, MO, USA) and anti-rabbit PLUS probe DUO92002 and antimouse MINUS probe DUO92004, both from Sigma-Aldrich (St. Louis, MO, USA).

For Western blot, we used the following primary antibodies: goat polyclonal anti-TRRAP (sc-5405, Lot #H150, dilution 1:1000), rabbit polyclonal anti-MAX mouse (sc-197, Lot #J0809, dilution 1:1000), mouse monoclonal anti-β-actin (C-4, sc-47778, Lot #D0618, dilution 1:3000), and mouse monoclonal anti-a-tubulin (DM1A, sc-32293, Lot #C0818, dilution 1:2000) from Santa Cruz Biotechnology (Dallas, TX, USA); rabbit polyclonal anti-c-MYC (#9402, Lot #11, dilution 1:2000) from Cell Signaling Technology (Danvers, MA, USA), and rabbit polyclonal anti-TBP (ab63766, Lot GR3319877-6, dilution 1:1000) from Abcam (Cambridge, UK). Secondary antibodies were horseradish peroxidase tagged anti-mouse, anti-rabbit, or anti-goat secondary antibodies (Cat No. P044801-2, P044701-2, or P0449, dilution 1:3000, Agilent Technologies, North Billerica, MA, USA).

Validation

All antibodies are commercially available and had been previously validated by the manufacturers. We tested them using positive controls, besides being compared to other antibodies for the same proteins in Western Blot and Immunofluorescence. In the Western blot, the band of each protein corresponded to its expected molecular weight as shown in the uncropped blots of our study.

Links to the data sheets of the antibodies, which include references, are found below. Additional references are included here when not listed in the manufacturer's webpage:

anti-c-MYC (9E10): https://www.scbt.com/sv/p/c-myc-antibody-9e10
anti-c-MYC (#9402): https://www.cellsignal.com/products/primary-antibodies/c-myc-antibody/9402
anti-MAX (sc-197): https://www.scbt.com/p/max-antibody-c-17
anti-TRRAP (SAB1300444): https://www.sigmaaldrich.com/SE/en/product/sigma/sab1300444#product-documentation
anti-TRRAP (sc-5405): https://www.antibodyregistry.org/AB_2209666. Additional references: PMID: 17967894, PMID: 29168693.
anti-TBP (ab63766): https://www.abcam.com/en-za/products/primary-antibodies/tata-binding-protein-tbp-antibody-nuclear-loading-control-and-chip-grade-ab63766#
anti-β-actin (sc-47778): https://datasheets.scbt.com/sc-47778.pdf
anti-a-tubulin (sc-32293): https://datasheets.scbt.com/sc-32293.pdf

Eukaryotic cell lines

Policy information about cell lines and Sex and Gender in Research

Cell line source(s)	Human neuroblastoma SH-SY5Y, female origin. These cells were provided as a gift by Professor Sven Pahlman, Lund University, Sweden.
Authentication	STR
Mycoplasma contamination	Mycoplasma testing performed and showing negative results.
Commonly misidentified lines (See ICLAC register)	There is no misidentified cells lines used in our study.
